# Amino acids and mammary gland development: nutritional implications for milk production and neonatal growth

**DOI:** 10.1186/s40104-016-0078-8

**Published:** 2016-04-02

**Authors:** Reza Rezaei, Zhenlong Wu, Yongqing Hou, Fuller W. Bazer, Guoyao Wu

**Affiliations:** Department of Animal Science, Texas A&M University, College Station, TX 77843 USA; State Key Laboratory of Animal Nutrition, China Agricultural University, Beijing, 100193 China; Hubei Key Laboratory of Animal Nutrition and Feed Science, Hubei Collaborative Innovation Center for Animal Nutrition and Feed Safety, Wuhan Polytechnic University, Wuhan, 430023 China

**Keywords:** Development, Health, Livestock, Mammary gland, Milk, Neonates, Production, Sows

## Abstract

Milk is synthesized by mammary epithelial cells of lactating mammals. The synthetic capacity of the mammary gland depends largely on the number and efficiency of functional mammary epithelial cells. Structural development of the mammary gland occurs during fetal growth, prepubertal and post-pubertal periods, pregnancy, and lactation under the control of various hormones (particularly estrogen, growth hormone, insulin-like growth factor-I, progesterone, placental lactogen, and prolactin) in a species- and stage-dependent manner. Milk is essential for the growth, development, and health of neonates. Amino acids (AA), present in both free and peptide-bound forms, are the most abundant organic nutrients in the milk of farm animals. Uptake of AA from the arterial blood of the lactating dam is the ultimate source of proteins (primarily β-casein and α-lactalbumin) and bioactive nitrogenous metabolites in milk. Results of recent studies indicate extensive catabolism of branched-chain AA (leucine, isoleucine and valine) and arginine to synthesize glutamate, glutamine, alanine, aspartate, asparagine, proline, and polyamines. The formation of polypeptides from AA is regulated not only by hormones (e.g., prolactin, insulin and glucocorticoids) and the rate of blood flow across the lactating mammary gland, but also by concentrations of AA, lipids, glucose, vitamins and minerals in the maternal plasma, as well as the activation of the mechanistic (mammalian) target rapamycin signaling by certain AA (e.g., arginine, branched-chain AA, and glutamine). Knowledge of AA utilization (including metabolism) by mammary epithelial cells will enhance our fundamental understanding of lactation biology and has important implications for improving the efficiency of livestock production worldwide.

## Background

**Fig. 1:**
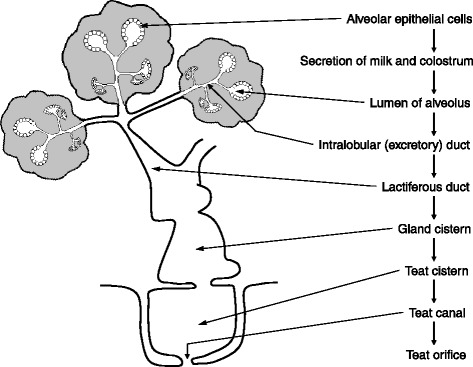
The basic structure of the mammary gland. Mammary epithelial cells are responsible for the synthesis and release of milk by lactating animals. The alveoli are connected to a duct system through which the secreted milk flows into the teat canal, from which it can be removed by suckling or milking

Milk consists of water, protein, lipids, carbohydrates (mainly lactose), minerals and vitamins, and is the secretory product of mammary epithelial cells (MEC) in all lactating mammals [1–62] (Table 1). The mammary gland is a compound, tubulo-alveolar structure with a merocrine/apocrine mode of secretion [63]. Proteins, some amino acids (AA), and fats are synthesized and secreted by MEC in response to hormones (e.g., prolactin, insulin and glucocorticoids) [64]. The alveoli are connected to a duct system through which the secreted milk flows into the teat canal from which it can be removed by suckling or milking [65] (Fig. 1). Thus, the lactating gland is a highly organized organ which supplies nutrients from the mother to the neonate through nursing.

**Table 1:** Composition of mature milk of domesticated and wild mammals

Species	Fat	Casein	Whey protein	Total protein	NPN subs	Lactose	Total Carb	Ca	Ash	DM^a^	References
Antelope^b^	72	48	14	62	18	42	47	2.6	13	212	[47, 48, 60]
Baboon	46	4.7	7.3	12	3.0	60	77	0.44	3.0	141	[5, 6, 20–24]
Bat	133	x	x	80	5.0	34	40	x	6.8	265	[26, 27, 29]
Bear (black)	220	88	57	145	7.5	3.0	27	3.6	19	419	[11, 24, 43, 58]
Bear (grizzly)	185	68	67	135	7.0	4.0	32	3.4	13	372	[11, 24, 48, 57]
Bear (polar)	331	71	38	109	5.6	4.0	30	3.0	12	488	[18, 21–24, 42]
Beaver	182	85	23	108	6.3	17	22	2.4	20	338	[3, 21, 40, 62]
Bison	35	37	8.0	45	3.0	51	57	1.2	9.6	150	[12, 35, 40]
Buffalo	77	38	7.0	45	5.2	40	47	1.9	8.0	192	[12, 35, 40, 48]
Blue whale	423	73	36	109	6.6	10	13	3.4	16	568	[16, 24, 42, 44]
Camel	45	29	10	39	5.6	49	56	1.4	7.0	153	[3, 12, 22, 40]
Cat (domestic)	108	31	60	91	10	42	49	1.8	6.2	264	[3, 21, 48]
Chimpanzee	37	4.8	7.2	12	2.0	70	82	0.36	11	144	[12, 22, 58, 60]
Cow (domestic)^c^	37	28	6.0	34	2.2	49	56	1.2	7.1	136	[19–24, 48, 58]
Coyote	107	x	x	99	x	30	32	x	9.0	247	[3, 22, 48]
Deer	197	94	10	104	14	26	30	2.6	14	359	[3, 12, 40, 48]
Dog (domestic)	95	51	23	74	23	33	38	2.0	12	242	[3, 20–24, 40]
Dolphin	330	39	29	68	3.0	10	11	1.5	7.5	420	[12, 40, 44, 49]
Donkey (Ass)	14	11	9.0	20	3.2	61	68	0.91	4.5	110	[3, 12, 20–24]
Elephant	116	19	30	49	4.1	51	60	0.80	7.6	237	[12, 20–24, 45]
Ferret	80	32	28	58	6.7	38	44	x	8.0	197	[6, 12, 21, 40]
Fin whale	286	82	38	120	6.2	2.0	26	3.0	16	454	[24, 31, 44]
Fox	63	x	x	63	4.0	47	50	3.4	10	190	[1, 2, 40, 48]
Giant panda	104	50	21	71	10	12	15	1.3	9.4	209	[38, 58]
Giraffe	125	48	8.0	56	2.2	34	40	1.5	8.7	232	[2, 12, 20, 40]
Goat (domestic)	45	29	5.0	34	5.8	43	47	1.4	7.9	139	[2, 24, 48, 52]
Goat (mountain)	57	24	7.0	31	5.3	28	32	1.3	12	136	[3, 12, 33, 40]
Gorilla	19	13	9.0	22	1.9	62	73	3.2	6.0	122	[12, 46, 61]
Guinea pig	39	66	15	81	12	30	36	1.6	8.2	176	[12, 36, 39, 40]
Hamster	126	58	32	90	11	32	38	2.1	14	279	[3, 12, 24, 60]
Horse (domestic)	19	14	8.3	22	3.6	62	69	0.95	5.1	119	[20–24, 48, 60]
Human^d^	42	4.4	6.6	11	2.8	70	80	0.32	2.2	138	[3, 12, 48, 60]
Kangaroo	21	23	23	46	4	<0.01	47	1.6	12	130	[3, 6, 40, 48]
Lion	189	57	36	93	6.6	27	34	0.82	14	337	[3, 9, 12, 40]
Llama	42	62	11	73	9.6	60	66	1.7	7.5	198	[12, 35, 37, 40]
Mink	80	x	x	74	12	69	76	1.3	10	252	[12, 29, 48, 59]
Moose	105	x	x	135	18	33	38	3.6	16	312	[8, 13, 23, 48]
Mouse (Lab)	121	70	20	90	11	30	36	2.5	15	273	[3, 12, 25, 48]
Mule^e^	18	x	x	20	3.0	55	62	0.76	4.8	108	[12, 20–24, 48]
Musk ox	110	35	18	53	7.0	27	33	3.0	18	221	[40, 48, 55]
Opossum	61	48	44	92	4.6	16	20	4.2	16	194	[15, 17, 30]
Peccary	36	40	15	55	5.7	66	71	1.2	6.4	174	[3, 48, 53, 60]
Pig (domestic)	80	28	20	48	5.4	52	58	3.1	9.2	201	[20–24, 48, 60]
Pronghorn	130	x	x	69	7.2	40	43	2.5	13	262	[3, 12, 48]
Rabbit	183	104	32	136	11	18	21	6.3	20	371	[3, 12, 40, 48]
Rat (Lab)	126	64	20	84	6.3	30	38	3.2	15	269	[3, 12, 48, 58]
Reindeer	203	86	15	101	14	28	35	3.1	14	367	[12, 20–24, 48]
Rhesus monkey	40	11	5.0	16	1.6	70	82	0.40	26	166	[14, 34, 48]
Rhinoceros	4.0	11	3.0	14	2.3	66	72	0.56	3.7	96	[20–24, 35, 48]
Sea lion	349	x	x	136	4.1	0.0	6.0	0.76	6.4	502	[44, 48, 50, 56]
Seal (fur)	251	46	43	89	6.9	1.0	24	0.70	5.0	376	[10, 42, 44, 56]
Seal (gray)	532	50	52	102	10	1.0	26	2.0	7.0	677	[42, 44, 48]
Seal (harp)	502	38	21	59	2.4	8.9	23	1.2	3.9	590	[7, 12, 40, 54]
Seal (hooded)	404	x	x	67	5.0	0.0	10	1.2	8.6	496	[12, 42, 44, 48]
Sheep (domestic)	74	46	10	56	2.7	48	55	1.9	9.2	195	[20–24, 40, 48]
Sperm whale^f^	153	32	50	82	6.3	20	22	1.5	8.0	270	[19, 44, 48]
Squirrel (gray)	121	50	24	74	16	30	34	3.6	12	257	[3, 12, 41]
Tree shrew	170	x	x	85	19	15	20	x	8.0	302	[3, 40, 60]
Water buffalo	74	32	6.0	38	5.8	48	55	1.9	7.8	181	[4, 6, 12, 35]
Water shrew	200	x	x	100	x	1.0	30	x	20	350	[3, 12, 20–24]
White whale	220	82	38	120	3.7	2.0	18	3.6	16	378	[12, 44, 48]
Wolf	96	x	x	92	4.8	32	35	4.0	25	253	[3, 12, 32, 60]
Yak	68	36	7.0	43	2.5	50	54	1.3	8.0	176	[6, 12, 35, 48]
Zebra	21	12	11	23	2.6	74	82	0.8	3.5	132	[35, 40, 48, 51]

Values are g/kg whole milk. Nonprotein nitrogen = total nitrogen – protein nitrogen. Nitrogen content in milk protein is 15.67 % [19–24]. The amount of non-protein nitrogenous substances (g/kg whole milk) is calculated as the amount of nonprotein nitrogen (g/kg whole milk) x 6.25. Caseins include *α*_S1_-casein, α_S2_-casein, β-casein, γ-casein, and *k*-casein. Whey proteins include α-lactoalbumin, β-lactoglobulin, serum albumin, immunoglobulins, lactoferrins, lysozymes, amino acid oxidases, xanthine oxidase, and other enzymes

*AA* amino acids, *Ca* calcium, *Carb carbohydrates*, *DM* dry matter, *NPN subs* non-protein nitrogenous substances (including free amino acids, small peptides, urea, ammonia, uric acid, creatine, creatinine, and other low-molecular weight nitrogenous substances). The symbol “x” denotes the lack of data in the literature

^a^Including fat, protein, NPN, lactose plus other carbohydrates, and minerals (total ash). When data on total carbohydrates have not been reported, ratios of lactose to other carbohydrates in milk are estimated to be 15:1 (g/g) [14, 38, 57, 58]

^b^Gemsbok antelope

^c^Concentrations of urea, creatinine, and amino sugars are 317, 127, and 392 mg/L whole mike, respectively

^d^Concentrations of urea, creatinine, and amino sugars are 274, 209, and 1111 mg/L whole mike, respectively

^e^Mule is a domesticated, hybrid animal produced by crossing a female horse with a male donkey

^f^Pygmy sperm whale

Milk is essential for the growth, development, and health of mammalian neonates. In livestock species (e.g., sows and cows), milk production is a limiting factor for maximum preweaning growth and survival of offspring [63, 64, 66]. Of note, nitrogen-containing substances (primarily β-casein, α-lactalbumin, other proteins, and free AA) are the most abundant organic nutrients in the milk of farm animals. Although fat and lactose syntheses in MEC have been extensively studied [48], there is a paucity of information regarding AA metabolism and milk protein synthesis in lactating mammals [67–69]. Branched-chain AA (BCAA; leucine, isoleucine and valine) and arginine are extensively degraded, whereas glutamate, glutamine, alanine, aspartate, asparagine, proline, and polyamines (key regulators of gene transcription and translation) are actively synthesized by lactating mammary tissue [67]. AA metabolism plays an essential role in milk production by regulating the maternal endocrine status, the rate of blood flow across the lactating mammary gland, and activation of the mechanistic (mammalian) target rapamycin (mTOR) signaling [70]. The major objective of this review article is to highlight recent advances in our understanding of mammary gland development and AA utilization for milk synthesis by MEC. Such fundamental knowledge is expected to enhance the efficiency of livestock production worldwide and to have important implications for human lactation.

## Significance of milk yield in the efficiency of livestock production

Mammals have a highly developed brain and complex body systems [63]. It usually takes a prolonged period of time for mammalian neonates to fully develop anatomically, physiologically and functionally. This developmental process occurs in utero during gestation and after birth. Prior to birth, the fetus is nurtured in a sterile, protected, moist, and warm environment of the uterus. The fetus is provided with nutrients and oxygen required for growth and development. At birth, the neonate is suddenly exposed to an environment with many potentially adverse situations, and also experiences a dramatic switch from primarily parenteral nutrition to exclusively enteral nutrition. This is associated with rapid changes in nutrient metabolism [70]. Because a newborn mammal has an underdeveloped ability to digest solid foods, it is greatly dependent on the mother’s liquid milk for nutritional support [63].

Milk composition varies among species (Table 1) or breeds within a species, stage of lactation, and different milking intervals [22, 24, 48]. Colostrum is the first secretion from the mammary gland immediately and within a few days after parturition. Colostrum is characterized by high concentrations of immunoglobulins necessary for conferring passive immunity in support of the underdeveloped immune system of the newborn [48]. Besides immunoglobulins and oligosaccharides [57, 58], colostrum and mature milk also contain nonnutrient substances [e.g., osteopontin, insulin-like growth factor I (IGF-I), and other bioactive factors] that are crucial for growth, development, health, and survival of the neonate [64]. Thus, adequate production of milk is essential to a high efficiency of livestock production. This is graphically illustrated by the pig industry where economic pressure drives an increase in production unit size [71]. The profitability of swine production is primarily determined by both the number and size of piglets produced per year per sow [72]. However, increased litter size results in lower birth weights for individual piglets [73]. The greatest proportion of mortality in commercial swine production occurs prior to weaning, with pre-weaning mortality ranging from 10 to 20 % of live-born pigs [73]. For example, pre-weaning mortality of piglets was 12.8 % in U.S. commercial swine herds during 2008 [74].

A variety of causes, including low birth weight and inadequate provision of milk, lead to high pre-weaning mortality and poor performance of livestock neonates [75]. For example, milk is the sole source of nutrients for suckling piglets, and their maximal growth performance and survival largely depend on sufficient production of milk by sows [73]. In turn, the weight achieved by the time of weaning determines adaptability of piglets to the nursery and how rapidly they reach market weight [74]. Indeed, secretion of milk by the mammary gland is the main determinant of neonatal growth rate; therefore, it is important that milk output by high-producing sows is adequate to allow maximum neonatal growth [76]. For instance, arginine (a nutritionally essential AA for neonatal pigs) is deficient in sow’s milk, which limits maximum growth performance of piglets [77]. In addition, the amount of milk produced by sows does not match energy and protein requirements of neonatal piglets (9 to10 pigs/litter) after d 8 of lactation. This gap gradually increases throughout lactation, so that maternal milk output meets only ~ 50 % of the needs of 21-day-old piglets for their maximum growth [76].

A sow must produce 18–20 kg milk/d to nurse a litter of 9 piglets, so that the neonates will have enough energy and protein to grow at a rate comparable to artificially-reared piglets of the same age [76]. In pigs, an average growth rate of at least 450 g/d from birth to 21 d of age (vs 230 g/d in sow-reared piglets) is achievable with an adequate supply of nutrients [76]. Similarly, calves nursed by lactating cattle do not grow at a maximal rate [63]. The biological basis for insufficient milk production by mammals (e.g., sows and cattle) is largely unknown. It may result from an inadequate supply of nutrients to mammary tissue, a deficit in endocrine stimulation of milk synthesis, and a lack of coordination in the metabolism of extra-mammary tissues (e.g., adipose, skeletal muscle, and small intestine) to spare nutrients for milk synthesis.

The pig is an animal model widely used in studies of lactation biology [71]. Sows have a high capacity to utilize nutrients from the arterial circulation for milk production [66]. However, due to inadequate feed intake by lactating sows, they do not meet physiological requirements for maximum milk production, thereby leading to a catabolic state in which their body reserves are mobilized to provide nutrients and energy for milk production during lactation [76]. Traditional feeding strategies for sows currently practiced in swine enterprises are unable to support sufficient milk production for optimal growth and survival of neonatal pigs [78]. Many studies have shown that high neonatal mortality and poor growth of pre-weaning pigs are highly correlated with insufficient milk production by sows [71]. Most of the studies have focused on hormonal regulation of lactation; however, little attention has been paid to the development of nutritional strategies that enhance lactogenesis in sows or the growth of suckling piglets [67].

## AA metabolism in the lactating mammary gland

AA are building blocks of proteins that regulate metabolic pathways for whole body homeostasis [70]. Lactating mammals require large amounts of AA to support milk synthesis by mammary glands during lactation [79]. Of nutritionally essential AA, branched-chain AA have received much attention in recent years [67–69]. For example, uptake of BCAA by the sow’s mammary glands is much higher than their output in milk, whereas the opposite is true for glutamine (Table 2) [69, 79, 80]. We have demonstrated that BCAA are catabolized extensively in lactating mammary tissue to provide amino groups for biosynthesis of other amino acids, such as glutamate and glutamine [69], which are necessary for neonatal growth and digestive tract maturation [67]. Recent studies have shown the important roles for BCAA in the regulation of mammary gland metabolism. For example, leucine increases protein synthesis in bovine MEC through activation of the mTOR cell signaling pathway [70].

**Table 2:** Uptake of amino acids by the mammary gland of the lactating sow and their output in milk on Day 14 of lactation

Amino acid	Uptake by the lactating mammary glands^a^	Output in milk	Difference
		g/day	
Arginine	31	6	+25
Proline	26	40	−14
BCAA	76	46	+30
Glutamine	16	36	−20

Adapted from Li et al. [69], Lei et al. [67], and Trottier et al. [80]

^a^Referring to a sum of all mammary glands

**Fig. 2:**
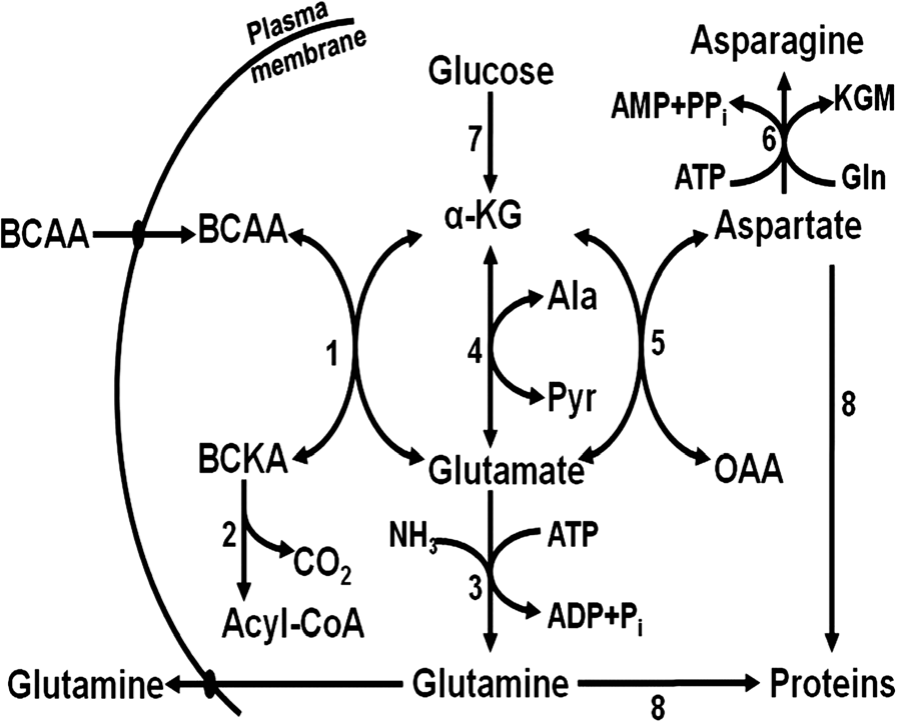
Pathways for BCAA catabolism and amino acid synthesis in lactating porcine mammary tissue. Enzymes that catalyze the indicated reactions are: (1) BCAT = branched-chain aminotransferase; (2) BCKAD = branched-chain alpha-keto acid dehydrogenase; (3) GS = glutamine synthetase; (4) GOT = glutamate-oxaloacetate transaminase; (5) GPT = glutamate-pyruvate transaminase; (6) AS = asparagine synthetase; (7) glucose metabolism via glycolysis and the Krebs cycle; (8) protein synthesis from glutamine, aspartate, alanine, asparagine, BCAA, and other amino acids. The corresponding a-ketoacids of leucine, isoleucine and valine are α-ketoisocaproate, α-keto-β-methylvalerate, and α-ketoisovalerate, respectively. Mammary tissue takes up BCAA and releases glutamine through specific transporters on the plasma membrane. This figure was reproduced from Li et al. [69] with permission from American Society for Nutrition

The overall pathway of BCAA catabolism in the lactating mammary gland is depicted in Fig. 2. The catabolism of BCAA by the mammary gland and MEC is initiated by BCAA transaminase in the presence of α-ketoglutarate to form branched-chain α-ketoacids (BCKA) and glutamate [69]. Thus, concentrations of BCKA in milk may be relatively high, and milk-born BCKA may serve as energy substrates for the small intestine of suckling neonates. In support of this view, there is evidence that substantial amounts of BCKA are utilized by enterocytes of the small intestine and the splanchnic bed of animals, including pigs, sheep and cattle [70]. The BCAA transaminase exists as mitochondrial and cytosolic isoforms. Both isoforms are expressed in MEC, and are activated by octanoate, a medium-chain fatty acid [68]. In studying the transamination of BCAA, their net flux through the BCAA transaminase should be measured [67], because this enzyme works at the thermodynamic equilibrium.

The BCAA-derived BCKA undergoes oxidative decarboxylation by the mitochondrial BCKA dehydrogenase complex (BCKAD) [70]. This enzyme complex consists of BCKA decarboxylase (E1 which requires thiamine pyrophosphate as a cofactor), dihydrolipoamide acyltransferase (E2 which requires lipoate and coenzyme A as cofactors), and dihydrolipoamide dehydrogenase [E3 which requires flavin adenine dinucleotide (FAD) and nicotinamide adenine dinucleotide (NAD) as cofactors]. The BCKAD E1 has two subunits: E1a and E1b. BCKAD is regulated by phosphorylation and dephosphorylation in cells. Protein levels for BCAT and BCKAD E1α, as well as dephosphorylation of BCKAD E1α are enhanced by leucine. Increasing extracellular concentrations of leucine from 0.5 to 2 mmol/L increased protein levels for mitochondrial BCAA transaminase and total BCKAD E1α by 39 % and 42 %, respectively, while decreasing the abundance of phosphorylated BCKAD E1α by 33 % in cultured bovine MEC [67]. As a result, the ratio of phosphorylated BCKAD E1α to total BCKAD E1α (PE1α/total E1α value) is reduced by 51 % in these cells [68]. Lactation increases rat mammary tissue BCAT activity by a factor of ten and sustains the BCKAD complex in the fully activated state [67]. For comparison, only 20 % of BCKAD exists in an active state in the mammary tissue of non-lactating rats [81]. Results of in vitro [69] and in vivo [80] studies indicate that the mammary gland is a quantitatively significant site for BCAA catabolism during lactation.

The BCAA-derived glutamate is either amidated to form glutamine or transaminated with pyruvate (or oxaloacetate) to produce alanine (or aspartate) in animal cells [69] (Fig. 2). Rates of synthesis of alanine, aspartate, asparagine, glutamate, and glutamine from BCAA in bovine MEC increase as the extracellular concentrations of leucine increase from 0 to 5 mmol/L, with the values being the highest for glutamine, followed by glutamate, aspartate, alanine, and asparagine in descending order [67]. Glutamine synthetase and phosphate-dependent glutaminase are the two key enzymes involved in glutamine synthesis and degradation, respectively, in most animal cells [70]. Interestingly, glutaminase activity is absent from the lactating mammary glands; therefore, MEC maximize the production and release of glutamine [69]. The *de novo* synthesis of glutamate and glutamine helps to explain the high abundance of these two AA in both free and peptide-bound forms in milk [82]. Accordingly, BCAA likely play an important role in milk synthesis by MEC. For example, approximately 30 g/d BCAA are degraded to form 20 g/d glutamine in the mammary glands of lactating sows (Table 2). The carbon skeleton of glutamine is derived primarily from glucose metabolism in MEC [69].

**Fig. 3:**
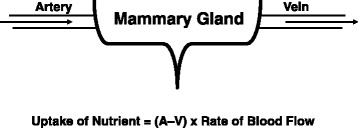
Nitric oxide-dependent blood flow across the mammary gland. Nitric oxide, which is synthesized from arginine in endothelial cells of the blood vessel, increases the rate of blood flow across the mammary gland by stimulating the generation of cGMP from GTP. cGMP activates protein kinase G in smooth muscle cells to promote their relaxation. Uptake of a nutrient by the mammary gland is calculated, based on the Fick principle, as the difference in the concentration of the nutrient between arterial and veinous blood × the rate of blood flow. A = concentration of a nutrient in arterial blood; V = concentration of the nutrient in venous blood

Although arginine is a nutritionally essential AA for piglets, it is markedly deficient in the milk of sows, cows, humans, and many mammals [70]. This results from the extensive catabolism of arginine by porcine mammary tissue [83]. For example, on Day 14 of lactation, uptake of arginine by the sow’s mammary gland is 31 g/d but the output of arginine in milk is only 6 g/d [79]. Thus, 81 % of the arginine taken up by the lactating gland from the arterial blood is degraded locally. In contrast, uptake of proline by the mammary gland is much lower than its output in milk (Table 2). Through enzymological and metabolic studies, we found that porcine mammary tissue express high activities of both type-I (cytosolic) and type-II (mitochondrial) arginase to hydrolyze arginine into ornithine and urea [83]. Most of the arginine-derived ornithine is converted into proline by ornithine aminotransferase and pyrroline-5-carboxylate reductase [70]. Mammary tissue does not contain pyrroline-5-carboxylate dehydrogenase or proline oxidase activity; therefore, this tissue cannot convert arginine, ornithine, or proline into glutamate or glutamine [83]. This helps explain the high abundance of proline in milk protein. Another metabolite of arginine catabolism is nitric oxide (a major endothelium-dependent relaxing factor), which enhances blood flow and, therefore, the uptake of nutrients by the lactating mammary gland (Fig. 3) [66]. Because of extensive degradation of arginine by arginase in MEC, inhibition of this enzyme may provide a new effective approach to beneficially enhance sow’s lactation performance and, consequently, piglet growth and survival.

## Structure of the mammary gland

### Classification of the mammary gland

Because the mammary gland is the foundation of lactation [63] and because AA are required for its function [67], it is important to highlight recent advances in our understanding of mammary gland biology. One of the major adaptations in mammals is the highly evolved mammary glands. A gland is a secretory organ whose products can be secreted into a cavity or directly into the blood in order to be distributed to tissues. The structure of glands can be simple (e.g., coiled tubular or branched alveolar), or compound (e.g., branched tubulo-alveolar). The mammary gland is an example of compound, branched tubuloalveolar gland [63]. Glands can also be categorized based on their mode of action: (a) apocrine (products are synthesized by the cells of the gland without causing their disintegration); (b) exocrine (a fluid is secreted and cells are not lost as part of the secretory process); (c) holocrine (secretion results from disintegrated cells of the gland); and (d) merocrine (the gland is repeatedly functional and cells are not destroyed during the secretory process). The secretions from the mammary gland take place via both merocrine and apocrine modes.

### Tissues of the mammary gland

In the lactating mammary gland, the parenchymal tissue is composed of epithelial structures (e.g., alveoli and ducts) and the associated stromal connective tissue. The stroma of a lactating gland is composed of connective tissue surrounding the epithelial structure [64]. The cellular components of the connective tissue consist of fibroblasts, blood vessels, and leukocytes, while non-cellular components include collagen and other connective-tissue proteins. In addition, an extensive white adipose tissue exists as part of the stroma of the developing gland (Fig. 1). This fat pad is considered as extra-parenchymal tissue, which is noticeably enlarged during the early phases of fetal and postnatal development but decreased progressively in mass during the periods of puberty, pregnancy, and lactation. Thus, the fat pad of the mammary gland is smaller during lactation than at the end of gestation.

Mammary glands are also considered accessory reproductive organs [65]. As noted previously, they are located on the ventral surface of the mammal. The alveolus is the basic milk secreting structure which consists of a single layer of secretory epithelial cells lining the alveolar lumen (Fig. 1). The epithelial layer is surrounded by a layer of contractile myoepithelial cells which play an important role in milk ejection. The alveolus is then surrounded by a basement membrane [64]. Upon stimulation of mechanoreceptors in the teat skin, these receptors induce cholinergic nerve impulses which travel via segmental pathways in the central nervous system to paraventricular nuclei and supraoptic nuclei in the hypothalamus, resulting in release of oxytocin from the posterior pituitary gland [48]. Oxytocin is a peptide consisting of nine AA and is produced in the paraventricular nuclei and supraoptic nuclei. Oxytocin is transported by carrier proteins (neurophysin I) from the cell bodies of paraventricular nuclei and supraoptic nuclei through the pituitary stalk. From the pituitary, oxytocin is released from neurophysin and enters the blood for transport to the mammary gland where it binds to its receptors (G-protein-coupled receptors) on the myoepithelial cells surrounding the alveoli. This causes myoepithelial cells to contract and force milk from the alveoli into the teat canal from which it is expelled via nursing or milking [84].

In pigs, the number of mammary glands varies from 14 to 20 (7–10 pairs). The arrangement of glands is in two parallel rows on either side of the ventral median line and extending from the pectoral to the inguinal region. Each gland is separate and independent of secretory tissue from adjacent glands. The heritability of teat number is rather low (0.10 to 0.20) [85]. Thus, it is difficult to select for high teat number in sows.

### Innervation in the mammary gland

The mammary gland is a skin-derived structure that contains external innervation in the skin covering the gland. There are few internal innervations which control the blood vessels in the mammary gland. Innervation inside the gland is sparse compared with other tissues. Sympathetic nerves are present in the mammary gland and are associated with the arteries, but not the alveoli. Sensory nerves present in the teat and skin are critical for initiating the afferent pathway (neural pathway) of the milk ejection reflex. There is no parasympathetic innervation of the mammary gland. This is similar to other skin glands. There is no innervation of the secretory system. Myoepithelial cells are not innervated but they contract in response to oxytocin [63].

### Blood and lymphatic vessels in the mammary gland

The blood supply to the mammary gland must leave and return to the body cavity through points where blood vessels exit the body cavity to reach the ventral portions of the skin, such as the thoracic and inguinal canal [86]. In pigs, blood supply to the mammary glands arises from two branches of the arterial system, the common carotid artery supplies the anterior glands and a branch of the abdominal aorta supplies the posterior glands. There is an anastomosis of the anterior and posterior mammary arteries and veins between the second and fourth inguinal glands such that the blood supplying the inguinal glands may pass forward through the anastomosis, whereas the blood supplying anterior glands may pass posteriorly through the anastomosis [64].

The extracellular fluids are drained from the mammary tissue and conducted back to the circulatory system via lymphatic vessels [64]. Also, the lymphatics contain leukocytes (particularly lymphocytes and macrophages) in lymph nodes, and these cells can mount an immune response to bacteria and foreign material. The lymphatic network serves to transport lipid-soluble nutrients (e.g., vitamin K and lipids) absorbed in the small intestine. The lymphatic capillaries are analogous to blood capillaries, but are much more permeable to large molecules than the blood capillaries. Note that lymph flow is unidirectional from tissues through lymphatic vessels into the veins [63].

## Development of the mammary gland

### Mammogenesis

**Fig. 4:**
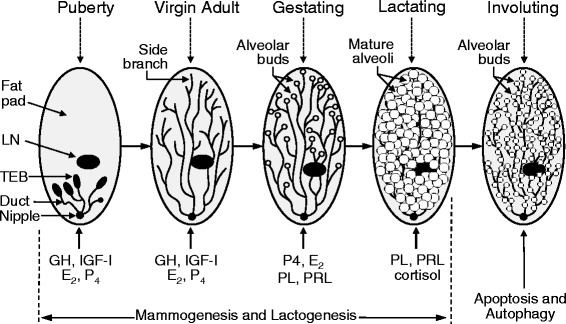
Development of the mammary gland in mammals. Mammogenesis begins during early fetal development. After birth, mammary ducts elongate through cell proliferation. At the onset of puberty, high concentrations of growth hormone, insulin-like growth factor in plasma stimulate mammary duct proliferation to form terminal end buds (TEBs) at the tips of the ducts. Under the influence of estrogen, TEBs actively proliferate to form ductal branches, which fill the mammary fat pad. After this stage, the TEBs regress. During pregnancy, progesterone and prolactin promote lobuloalveolar development to form alveolar buds. At the onset of lactation, mature alveoli capable of producing and secreting milk are formed. Suckling of the nipple by the neonate results in the contraction of the myoepithelial cells around the alveoli, causing the milk to be ejected through the ducts into the nipple. Upon weaning, lactation stops and the mammary gland undergoes involution through apoptosis and autophage to its nonlactating state. E2 = estrogen; GH = growth hormone; IGF-I = insulin-like growth factor-I; LN = lymph node; P4 = progesterone; PL = placental lactogen; PRL = prolactin

The mammary gland is one of a few tissues in mammals which can undergo repeated cycles of growth, functional differentiation, and regression (Fig. 4). The development of mammary gland structures is referred as mammogenesis, which begins during early fetal development and proceeds beyond the initiation of lactation. Most of the mammogenesis event occurs during gestation, and some pregnancy hormones such as estrogen and progesterone are responsible for mammary growth and maturation [87].

Differences among species in how lactation fits into the overall reproductive cycle is one of many interesting aspects of lactation biology. For example, a sow typically is bred to farrow for the first time at about 12 months of age (gestation is about 114 d). The majority of mammary development occurs during this 114-day period. Lactation begins at farrowing. Usually, the piglets are weaned at 21 d of age, when the mammary gland of the sow undergoes involution and milk secretion ceases [48].

Many mammalian species exhibit lactational anestrus; namely, they do not experience estrous cycles during early lactation. The length of lactational anestrus varies among species. For example, in the sow, gestation is 114 d and the piglets suckle the sow for several weeks (generally 3–4 wk) before weaning. Lactation in the sow inhibits her normal estrus and the next estrus occurs several days after the piglets are weaned. In fact, this management approach is used to synchronize estrus and breeding for the next farrowing. Under modern management procedures, lactation represents only one part of the entire reproductive process, but is nevertheless a critical period for neonatal survival and growth [88].

Lactation and reproductive processes are closely intertwined in mammals. Mammary development occurs only slightly during estrous cycles, while substantial development takes place while the female is pregnant and preparing to provide extra-uterine nutritional support to the newborn. Some of the factors involved in the maintenance of pregnancy also play an important role in regulating mammary gland development during pregnancy. For example, key pregnancy hormones, estrogen and progesterone, are the primary stimulators of growth of the mammary tissue. In addition, growth hormone, prolactin and glucocorticoids (mainly cortisol) contribute to mammary gland development. Similarly, important hormonal changes that occur in association with parturition are critical to the initiation of milk synthesis and secretion by the mammary tissue at birth [89].

During pregnancy, the mother is connected to her fetus via the placenta in utero. After birth, their interrelationships occur outside of the uterus via production of milk by the mammary gland. During lactation, the mother undergoes substantial changes in metabolism to account for the increased demands of milk synthesis. From this perspective, the mammary gland replaces the placental-uterine unit in providing nutrients and protective factors for development of the offspring [63]. With the evolution of the placenta resulting in extensive intrauterine development of the conceptus, the ability to lactate after expulsion of the offspring from the uterus becomes a critical part of the reproductive strategy of mammalian species.

Most of our understanding of the physiology of lactation (including structural development of the mammary gland and the control of nutrient supply to the mammary gland) has been acquired from studies of the dairy cow [89]. That is because of the combined importance of efficient milk production and the consumption of milk by humans for growth and health. Another reason is the commercial availability of milking equipment that allows for precise and easy measurement of total milk output by the cow to assess her mammary gland metabolism [79].

Structural development of the mammary gland generally takes place in five phases. These phases are the fetal period, the prepubertal period, the post-pubertal period, pregnancy, and lactation [63]. The postnatal development of the mammary gland is outlined in Fig. 4. The associated morphological changes, which depend on the adequate provision of AA [64, 67], are distinct and have been well characterized in cows and mice. Here, we will review hormonal regulation of the development of the mammary gland.

### Hormonal regulation of mammary gland development

#### During the fetal period

Testosterone injection into the pregnant mouse on d 8, 9, or 10 of pregnancy leads to masculinization of the mammary gland of female fetuses. The female fetuses are born without nipples. The mammary bud becomes exteriorized and is separated from the epidermis, as observed by d 14 or 15. Mammary development in male fetuses is not further affected by the testosterone treatment of the mother. Gonadectomy of male fetuses results in the female pattern of mammary development in male fetuses. Gonadectomy resulted from localized irradiation on d 13 of pregnancy of the region where the gonads form in the mouse fetus. This functionally castrates the fetus. By d 18 of pregnancy, mammary development in male mice includes formation of normal primary mammary cords. Gonadectomy of the female fetus by irradiation does not alter normal mammary development [90]. Administration of high doses of estrogen to female fetuses or to the mother results in abnormal mammary development [91]. The abnormalities include total suppression of development of the mammary bud, leaving only a nipple with no internal structures; partial inhibition of the mammary bud; formation of a cavity at the site of the mammary bud; abnormally shaped mammary buds; formation of multiple primary mammary cords; and excessive formation of the mesenchyme around the primary mammary duct. Likewise, administration of growth hormone (somatotropin) into the mouse fetus increases the size of the developing mammary gland in both male and female fetuses, suggesting that this hormone is also involved in fetal development of the mammary gland [92].

#### During the pre-pubertal period

Daily injections of growth hormone to heifers from 8 to 15.6 months of age resulted in increased mammary parenchyma and decreased extraparenchymal tissue compared to controls [93]. Growth hormone stimulates mammary growth through increasing the hepatic synthesis of insulin-like growth factor-1 (IGF-1), which is a potent mitogen for MEC [64]. Therefore, growth hormone may be a major factor controlling mammary gland development during the prepartum period. However, administration of growth hormone during the prepubertal period does not increase milk yield during the first lactation period [92].

Leptin, which is produced by white adipocytes, is another protein that influences mammary development indirectly and this may explain why excessive fattening impairs mammary development. For example, results from both in vitro and in vivo studies indicate that leptin decreases IGF-1-induced proliferation of bovine MEC [63]. However, leptin does not mediate the effects of a high-plane of nutrition on MEC in prepubertal heifers due to the paucity of leptin receptors on those cells. It appears that leptin exerts its effects on mammogenesis through inhibition of IGF-1-mediated cell signaling in MEC that is linked to activation of macrophages in the mammary gland. Upon stimulation with leptin, macrophages increase their phagocytic activity and production of cytokines such as TNFβ and IL-6, which lead to inhibition of IGF-1-induced proliferation of many cell types [94].

#### During the post-pubertal period

The growth of mammary tissue during estrus is related to ovarian steroid hormones [64]. Estrogen receptors and progesterone receptors both appear in cells of the mammary gland around the time of puberty. However, the exact roles of estrogen and progesterone are not completely understood. Mammogenic hormones establish the conditions for specific growth patterns in mammary tissue [63]. For example, concurrently elevated concentrations of estrogen and progesterone in plasma during late gestation result in an exponential increase in parenchymal growth and in the formation of alveoli. In contrast, cyclic changes in those hormones associated with estrous cycles result primarily in duct elongation and formation of some lobular tissue, but not in formation of alveoli. Thus, mammary gland development is usually driven by a complex of hormones acting in concert.

Effects of many mammogenic hormones are thought to be mediated through stromal cell-derived growth factors which act in a paracrine manner by eliciting mitogenic responses in the adjacent epithelial cells. Much of our current understanding of how mammogenic hormones and growth factors function arises from research in rodents. However, similarities are being recognized in other mammals [63]. As noted previously, estrogen is an important mammogenic factor, particularly in the postpubertal female. Estrogen receptors (particularly estrogen receptor α) appear in the gland around the time of puberty, coinciding with the period when the gland becomes exposed to cyclic increases in concentrations of estrogens in blood. In rodents, estrogen acts via its receptors in the stromal tissue to stimulate production of growth factors, which in turn stimulate ductal development. The evidence available from studies of cattle also indicates that the effect of estrogen on enhancing mammary gland development may be mediated through the stroma [95].

Progesterone is another ovarian steroid hormone that plays a key role in mammary gland development. While progesterone receptors have been difficult to identify in the mammary fat pad, administration of progesterone stimulates proliferation of stromal cells under physiological conditions [63]. The stimulatory effect of progesterone on DNA synthesis in ductal epithelium is probably mediated indirectly through its effects on stromal cells. The major mammogenic effects of progesterone require its receptors in MEC and include ductal side branching or alveolar bud formation, which are the hallmarks of post-pubertal mammary gland development. Estrogen stimulation of progesterone receptor expression in MEC is necessary for progesterone to exert its effects. Therefore, progesterone has a major role in alveolar morphogenesis but a lesser role in ductal morphogenesis. During estrous cycles, duct elongation and expansion of the parenchymal tissue into the fat pad occur when the circulating concentrations of estrogen are high. During the luteal phase when concentrations of progesterone in plasma are greatest, little growth of the mammary gland occurs in ruminants, but the formation and maintenance of lobular structures in the mammary gland may be stimulated by progesterone, with little regression of the mammary ductal system occurring between estrous cycles [64].

Synergy between estrogen and progesterone is observed during pregnancy when both hormones are present in high concentrations. Elevated concentrations of estrogen and progesterone in blood establish the conditions required for exponential growth of the mammary gland during pregnancy [95]. Lobulo-alveolar development represents the greatest increase in mammary tissue mass during pregnancy. In the cow, circulating concentrations of progesterone are elevated throughout gestation, while circulating concentrations of unconjugated estrogens are greatest during the later stages of gestation when there is the greatest increase in mammary tissue mass. Estrogen and progesterone have direct effects on the mammary gland, probably mediated via autocrine and paracrine factors produced locally in the tissue. In addition, steroid hormones may have indirect effects on the secretion of prolactin by the anterior pituitary gland [76].

Prolactin is required for initiation of lactation and galactopoeisis, but this hormone also has a mammogenic effect. Prolactin receptors are present in the mammary fat pad and the mammary gland epithelium. Prolactin may act on both epithelial and stromal components of the growing mammary tissue. Inhibition of prolactin secretion retards mammary gland development in pregnant goats, pigs and other species [96], although concentrations of prolactin in plasma are normally low during pregnancy [63]. There is evidence that mammary gland development during pregnancy may not be limited by low circulating levels of prolactin [96]. The action of prolactin may be regulated by estrogen-induced changes in expression of receptors for prolactin in mammary tissue [96].

Administration of growth hormone to cattle is known to stimulate milk production during lactation. This effect is indirect in that growth hormone enhances secretion of IGF-I from the liver, which in turn mediates many of the galactopoeitic effects of growth hormone during lactation [97]. Growth hormone also acts as a mammogenic hormone and can stimulate mammary gland growth at all stages of development. Direct effects of growth hormone on mammary tissue require the presence of growth hormone receptors on MEC. While this remains a point of controversy, there are reports that growth hormone receptors are expressed in MEC or mammary stromal cells in various species [63, 64]. Several lines of evidence indicate that growth hormone may act on mammary tissue in ruminants by stimulating stromal production of IGF-I which is mitogenic for MEC [97]. The highest level of IGF-I expression in mammary tissue occurs in the fat pad and is greatest during the prepubertal growth phase and during late pregnancy. Expression of IGF-I in mammary tissue is regulated by growth hormone, estrogen, and positive feedback stimulation from proliferating MEC. The function of IGF-II in mammary gland growth is less clear than for IGF-I. Stromal cells probably produce IGF-II, which is regulated by the stage of mammary development and hormonal stimulation [63].

Placental lactogens are secreted by the placenta and may have prolactin- or growth hormone-like activities, depending upon the species. In pregnant goats, concentrations of placental lactogen in maternal blood is closely correlated with the number of fetuses present and, therefore, placental mass [64]. Placental lactogen, in combination with other mammogenic hormones, may regulate the extent of mammary development during late pregnancy. In dairy cows, there is a relationship between placental mass and subsequent milk production [97]. However, the concentration of placental lactogen in maternal blood of dairy cows is low and the effect of placental mass may result from other placental hormones, including estrogen [98]. Other hormones also are required for mammary gland growth, including glucocorticoids, thyroid hormones and insulin [97]. For example, severely diabetic mice treated with estrogen and progesterone will develop extensive lobuloalveolar structures. Of note, insulin synergizes with estrogen and progesterone to increase mammary gland development. Normal concentrations of insulin in blood is not limiting for normal mammary development [98].

#### During pregnancy

##### Estrogen and progesterone

Optimal mammary growth requires both estrogen and progesterone. During pregnancy, both MEC and mammary stromal cells express estrogen receptor alpha and progesterone receptors. During lactation, MEC express estrogen receptor alpha, but no progesterone receptors. In fact, the abundances of progesterone receptors are inversely proportional to the secretory activity of the gland. Concurrently, changes in circulating levels of estrogen and progesterone during pregnancy establish conditions for proliferation of MEC and lobuloalveolar growth, which is characteristic of mammary tissue development during pregnancy. In pigs, on d 30 of pregnancy, elevated estrogen levels signal to increase DNA concentrations in mammary glands, which continue to grow throughout pregnancy with a peak between d 75 and 90 [99]. In cows, progesterone is elevated throughout gestation (required for maintenance of pregnancy), while estrogen is markedly elevated during the second half of gestation [97]. Consequently, most mammary growth during the first half of gestation is ductal growth and lobular formation, whereas in the second half of gestation, ductal growth continues, with most growth occurring in lobuloalveolar cells [63]. In hypophysectomized-ovariectomized goats, administration of estradiol, progesterone, prolactin, growth hormone, adrenocorticotrophic hormone (ACTH) and glucocorticoids (particularly cortisol) are required for lobuloalveolar development comparable to that in mid-pregnancy [63]. This suggests that all of these hormones are involved in mammary gland development during pregnancy. The roles of some of these and other hormones in mammals are discussed below.

##### Prolactin and growth hormone

In virgin rats, transplantation of the pituitary to the kidney capsule releases the anterior pituitary lactotrophs from inhibition of secretion of prolactin by dopamine (a metabolite of tyrosine), which results in marked stimulation of growth of the mammary gland. However, circulating levels of both prolactin and growth hormone are normally reduced during gestation in most species [63]. Although both prolactin and growth hormone are required for mammary gland development, their concentrations in blood are not normally limiting. Growth hormone is implicated in mammary gland growth during fetal and prepubertal stages [76].

##### Placental lactogen

Placental lactogens are synthesized and secreted from the chorion of the placenta. Generally they have both prolactin- and growth hormone-like activities. However, there is great variation among species. Pigs and rabbits do not have placental lactogen [94]. In rats, during the first half of pregnancy, prolactin and growth hormone are the primary hormones involved in mammary gland development, and significant amounts of placental lactogen are not secreted until d 12 of gestation [100]. Interestingly, the hypophysectomy of the rat at d 12 of pregnancy or later, does not affect mammary cell numbers or maintenance of pregnancy [100]. In goats, the concentration of placental lactogen in maternal blood is closely correlated with the number of fetuses [101]. One interpretation of this observation is that the level of total lactogenic hormone activity in maternal blood (prolactin plus placental lactogen or other hormones) regulates the growth of mammary glands during late pregnancy. However, in cows, concentrations of placental lactogen in maternal blood are very low [97], and this hormone may synergize with estradiol, progesterone, prolactin and growth hormone to promote mammary gland development [63]. It should be borne in mind that the placenta secretes estrogens and progesterone in a highly species-dependent manner [98].

##### Relaxin

Relaxin is a hormone secreted during pregnancy in some species. Its role is to soften the cervix and prepare the reproductive tract for parturition. Most effects of relaxin require estrogen stimulation [63]. Relaxin also has a major mammogenic role in sows. Removal of the ovary from late pregnant gilts (at d 80 or d 100 of pregnancy in association with administration of exogenous progesterone to maintain pregnancy) removes the source of relaxin in gestating swine [102]. This led to a substantial reduction in mammary gland development during the last weeks of pregnancy. Of note, although pigs do not produce placental lactogen, the corpora lutea do secrete relaxin as a major mammogenic factor during pregnancy [64]. The concentration of relaxin in the maternal blood is positively correlated with the number of corpora lutea in the ovaries in pigs. Additionally, the number of corpora lutea is directly related to the number of ovulated oocytes and the number of fetuses. Thus, there is a relationship between the number of fetuses and the level of mammogenic stimulus on development of the mammary gland in pigs [102].

##### Insulin

Insulin is required to maintain normal mammary tissue function. Mammary epithelial cells are resistant to insulin during mid and late gestation periods, exhibit higher sensitivity to insulin during lactation, and become insensitive again during involution of the mammary gland [103]. Insulin stimulates mitosis of MEC in vitro, but this action of insulin is not essential for mammary development in vivo [104]. Concentrations of insulin in blood decrease during gestation as do concentrations of prolactin and growth hormone; therefore, it can be surmised that insulin is not a major factor directly affecting mammary development during pregnancy [96].

##### Thyroid hormones

Thyroid hormones enhance metabolic rates and oxygen consumption by animals. Their effect on mammary gland development is probably indirect or via the normal requirements of cell maintenance [64]. Hypothyroidism retards ductal and lobuloalveolar growth in rat mammary tissue, but administration of thyroid hormones restores the normal developmental pattern [98].

#### During lactation

The number of cells in the lactating mammary gland is critical for milk production and they continue to increase after parturition. Mammary tissue weights and total DNA content continue to increase in early lactation. The impact of this increased mass of mammary tissue on milk production can be substantial in some species. For example, the total amount of DNA in mammary glands of rats during lactation is highly correlated with litter weight gain [98]. In rats, total mammary DNA can increase by over 100 % during lactation, depending upon litter size. In sows, mammary gland weight and total mammary DNA increase by 55 % and 100 %, respectively, between d 5 and 21 of lactation when the sow nurses 9 or 10 piglets [105]. In cows, mammary DNA increases by 65 % from d 10 prepartum to d 10 postpartum, although how much of this increase occurred pre- and postpartum was not determined [63]. At present, little is known about cell numbers in the bovine mammary gland throughout the lactating period.

Litter size in some species (e.g., the rat or pig) is directly related to nursing intensity (a combination of number of the nursing neonates and the intensity with which they nurse). Nursing intensity and parity have major effects on the extent of mammary gland growth during lactation [102]. There is considerable evidence that mammary growth is enhanced by nursing and that the mammary tissue of the lactating sows has substantial growth potential [96].

## Autocrine and paracrine regulation of mammary gland growth

Autocrine and paracrine factors (local growth factors) play a major role in growth of the mammary glands. Many effects of steroid hormones on growth of the mammary gland are mediated by local growth factors and those effects include an interaction between MEC and the mammary fat pad. MEC will grow and remodel when transplanted into the fat pad. The interaction may involve specific fatty acids from the fat pad that induce changes in proliferation and differentiation of MEC [106]. Stromal cells in the region of the terminal end buds or the terminal ductal lobular unit of mammary glands may help degrade collagen so that their structures can expand. The enlarging ducts may promote mesenchymal growth and angiogenesis. Epithelial cells in the terminal ductal lobular unit and developing ducts probably interact with each other to induce synthesis and assembly of the basement membrane [63].

As noted previously, a number of other growth factors besides IGF-1 have positive or negative effects on mammary gland development [97]. Local production of transforming growth factor-ß (TGFB) inhibits mammary growth, such as during the prepubertal period and between estrous cycles. In contrast, epidermal growth factor (EGF) and TGF-alpha (TGFA) produced in the mammary tissue stimulate MEC proliferation [63]. Both EGF and TGFA bind to EGF receptors. The mammogenic action of estrogen and progesterone occurs in part through decreasing local production of the inhibitory TGFB, while increasing local production of TGFA and expression of EGF receptors in MEC. EGF receptors in mammary stromal cells are also necessary for normal ductal growth [107]. Other growth factors, which are produced by stromal cells as epithelial cell mitogens, include hepatocyte growth factor and members of the fibroblast growth factor (FGF) family (e.g., acidic FGF and FGF7). Basic FGF is also an epithelial cell mitogen, although its origin in mammary tissue remains uncertain.

Fatty acids, particularly unsaturated fatty acids, stimulate MEC growth and enhance effects of other growth factors such as IGF-I and EGF [106]. As noted previously, mammary stromal cells contribute to dissolve the extracellular matrix of the mammary gland so that the epithelial structures can continue to grow. Several proteases play roles in the remodeling and growth of the parenchymal tissue. Extracellular matrix components (e.g., proteoglycans, hyaluronan, fibronectin, and laminin), which are important for mammary gland growth and function, are produced by both epithelial cells and stromal cells [94].

## Lactation in mammals

Lactation is a critical component of the reproductive strategy of mammals and is intimately intertwined with the physiology of reproduction. Lactation is defined as the combined processes of milk synthesis, secretion and removal. Milk secretion refers to the release of milk by MEC and the passage of milk into the lumen of the alveoli of the mammary gland. Milk removal is the passive removal from the cisterns after the ejection of milk from the lumen of the alveoli to the milk duct [63].

### Cycles of lactation

Mammals reproduce more than once, and therefore, lactate more than once. The mammary gland is one of the relatively few structures of the body which undergoes repeated cycles of structural development, functional differentiation, and regression. The cycle of lactation includes four events. First, the mammary gland undergoes mammogenesis, with the most dramatic change in its structural development occurring during pregnancy. Second, synthesis and secretion of colostrum begin immediately after parturition. Third, synthesis and secretion of mature milk are maintained for weeks and months until the young no longer need milk or milk is no longer removed from the gland. Fourth, involution of the mammary gland follows the cessation of milk secretion and the cycle of lactation will start again with stimulatory effects of the hormones of new pregnancy and peri-parturient events. These phases of mammary development are at the center of lactation biology. In essence, they include the onset, maintenance and termination of lactation.

### Onset of lactation

The onset of lactation can be divided into two stages: Stage 1 and Stage 2 lactogenesis. In pigs, Stage 1 lactogenesis occurs between d 90 and d 105 of pregnancy, and Stage 2 lactogenesis is between d 112 of pregnancy and early lactation which is concomitant with copious secretion of milk. By d 4 of lactation, MEC are fully differentiated [99].

Lactogenesis consists of a series of events in the cell differentiation process whereby MEC are converted from a nonsecretory to a secretory state. Stage 1 consists of cytologic and enzymatic differentiation of alveolar cells and coincides with limited milk secretion before parturition. The prelactating alveolar epithelial cells have an irregular shaped nucleus, minimal rough endoplasmic reticulum (RER), a small inconspicuous Golgi complex, few microvilli at the apical surface, few mitochondria, and perhaps 1 or 2 fat droplets. Immediately before and during parturition, there is dramatic hypertrophy of the RER, enlargement of the Golgi, appearance of large vesicles containing casein micelles, release of granular material (casein micelles) into the lumen, an increase in the number of cytoplasmic fat droplets and their release into the lumen, an increase in the number of microvilli at the apical cell membrane, and an increase in the number of mitochondria per cell. Cell polarity becomes evident: the RER is primarily in the basal half of the MEC, the nucleus is shifted to the basal portion of the cell, and the Golgi is apical to the nucleus [64].

Enzymatic changes include increased expression of acetyl-CoA carboxylase, fatty acid synthetase, and other enzymes associated with lactation, as well as increases in the activities of transport systems for AA, glucose, fatty acids, and other substrates for milk production. Synthesis of α-lactalbumin, and therefore, lactose, does not begin until Stage 2 of lactogenesis. The mammary glands enter Stage 1 lactogenesis days or weeks (e.g., five weeks in pigs) prior to farrowing. This stage may be detected by measureable amounts of colostral proteins in blood. Colostrum production takes place during Stage 1 lactogenesis, when high levels of progesterone in plasma inhibit lactation [63].

At farrowing, concentrations of progesterone in plasma decrease rapidly which relieves inhibition of milk synthesis, while increasing concentrations of prolactin in the plasma stimulate synthesis of milk components (e.g., α-lactalbumin and lactose) and growth of the mammary gland [102]. These changes are probably the main driving forces for the mammary gland entering Stage 2 lactogenesis characterized by initiation of copious milk production, which begins 0 to 4 d before parturition and extends through a few days postpartum in swine. Other hormones such as estrogen, oxytocin and relaxin are also involved in this process [63]. Suckling of the mammary gland by piglets is an additional factor for initiating the onset of Stage 2 lactogenesis. This is different from other species such as cows and humans where milk production is initiated irrespective to whether the glands are being suckled or not [96].

### Maintenance of lactation

In Stage 2 lactogenesis, milk removal is necessary to maintain milk production by mammary glands. If milk removal ceases, such as when a piglet dies, that mammary gland undergoes rapid involution. Involution is induced if milk stasis occurs for an extended period of time, often less than one day. Milk production cannot be restored in individual glands after the involution process reaches a “point of no return” after 40–60 h of milk stasis [96].

However, during the next reproductive cycle, all glands start to re-develop and they will again produce milk if suckled after parturition. Development of mammary glands and their synthesis of milk are regulated at the gland level by interactions between systemic factors (e.g., circulating hormones and nutrients) and local factors (e.g., hormone receptors on the membranes of MEC). Immediately after milk removal, systemic factors, of which prolactin is the most important, are responsible for stimulating milk production. The rate of milk synthesis is high during the first 30–35 min after a suckling bout. As filling of the mammary gland progresses, milk synthesis is inhibited by local factors (e.g., hormone receptors and transcription factors) controlled by milk stasis [108].

When the mammary gland is emptied once again at the next suckling, the inhibition of milk synthesis due to milk stasis is relieved and milk synthesis resumes at the maximum rate [102]. A lactogenic complex consisting of insulin, glucocorticoids and prolactin is responsible for maintenance of lactation in many species. In pigs, in contrast to the onset of lactation, maintenance of lactation is dependent on piglet suckling and prolactin [109]. Suckling by piglets induces release of prolactin from lactotrophs in the anterior pituitary gland into the blood, and upon binding to receptors in MEC, prolactin elicits signals to stimulate milk production and mammary tissue growth [63].

Prolactin plays an important role in milk production by sows [96]. When sows were given bromocriptine to inhibit prolactin release, there was a marked reduction in milk yield, whereas milk production increased again when the bromocriptine treatment ceased. However, the concentration of prolactin in plasma is normally not the limiting factor for milk production, and improvements in milk yield of sows using exogenous prolactin treatment has been attempted without success. No other hormones are known to be involved directly in maintaining lactation [102]. The hormone oxytocin is clearly necessary for milk letdown, but there is no knowledge of its direct role in maintaining milk synthesis [63]. In contrast, growth hormone has a well described lactogenic effect in dairy cows, but administration of porcine growth hormone to lactating sows did not influence milk yield [109].

### Termination of lactation

Mammary glands begin to involute rapidly if not suckled. This occurs in early lactation for mammary glands when the young (e.g., piglets) develop a teat preference or at any time throughout lactation if a neonate (e.g., a piglet) dies and thus its associated mammary gland is not suckled [102]. Involution of the mammary gland occurs immediately follows weaning and is characterized by apoptosis or loss of the alveolar epithelial cells and proteolytic degradation through autophagy [89]. Rates of epithelial cell apoptosis vary with species [63]. Apoptosis, usually caused by DNA fragmentation, is thought to be correlated with decreases in circulating concentrations of prolactin, growth hormone, and IGF1 [89]. There is no fundamental difference between involution of individual mammary glands during lactation or after weaning [109]. If milk stasis remains in one or more glands for one-half day, expression of prolactin receptors in the mammary gland decreases, thereby uncoupling the stimulatory effects of prolactin on milk synthesis [96]. Furthermore, milk stasis induces a mechanism that leads to programmed cell death or apoptosis. Once started, apoptosis has severe consequences on the mammary gland. If milk stasis is relieved after 24 h, milk yield is reduced by approximately 25 % throughout the remaining period of lactation. If milk stasis is not relieved within 50–60 h, the mammary gland will become totally involuted and nonfunctional [102].

## Synthesis of milk by MEC

Analyses of the composition of milk revealed not only macro- and micro-nutrients but also a variety of protective factors, including antimicrobial lipids and proteins in milk (Table 1). Most of the protective factors are not specifically produced by the mammary gland itself, but instead they are directly taken up from the mother’s blood and transferred into milk. Milk proteins include casein, lactalbumin, osteopontin, relaxin, as well as lactoferrin which binds iron to prevent growth of microorganisms, lysozyme which hydrolyzes bacterial cell walls, lactoperoxidase which oxidizes bacterial components, and secretory immunoglobulins, IgA and IgM. These secretory factors are effectively involved in protecting the gastrointestinal tract from damage induced by environmental insults [96]. The levels and activities of these factors are species specific. For instance, bovine milk contains low concentrations of taurine, lactoferrin, lysozyme, and IgA, but relatively high concentrations of lactoperoxidase. In contrast, human milk has higher concentrations of taurine, lactoferrin, lysozymes, IgA, and antimicrobial activity, but lower concentrations of lactoperoxidase than bovine milk [63].

Milk is an emulsion of fat globules and a suspension of casein micelles (casein, calcium, and phosphorous) in an aqueous phase that contains solubilized lactose, whey proteins, and minerals. The maternal blood is the ultimate source of all nutrients needed for milk synthesis by MEC. Specifically, the precursors of milk components leave the blood into the extracellular fluid and then enter MEC through the basolateral membrane of the cell. In addition, some pre-formed proteins (e.g., immunoglobulins) and AA (e.g., BCAA and taurine), are transported intact across MEC and incorporated into milk [48, 67].

### Synthesis of proteins

Amino acids present in the maternal blood circulation are taken up by the MEC through specific AA transport systems on its basolateral membrane. Once inside the cell, AA are covalently bound together via peptide bonds to form proteins in the poly-ribosomes on the RER. Proteins that are synthesized at the RER are either secreted proteins (e.g., casein, β-lactoglobulin, and α-lactalbumin), membrane-bound proteins (e.g., extra-cellular matrix proteins involved in cell-cell contacts and membrane-bound enzymes), or intracellular proteins. Newly synthesized proteins are transferred from the RER to the Golgi apparatus where they are processed for transport out of the cell. Casein is secreted as a micelle, which is formed in the Golgi from casein, calcium and phosphorous. Proteins that remain in MEC include cellular enzymes, structural proteins (e.g., keratin), and fatty acid-binding proteins [63].

Milk proteins and lactose are transported to the apical area of the MEC via secretory vesicles that bud off of the Golgi. These secretory vesicles are bounded by a lipid bilayer membrane and make their way to the apical membrane via a mechanism involving microtubules made of polymerized tubulin [48]. Tubulin is one of several cytoskeletal proteins which form the cellular scaffolding, providing the cell with structure. Keratin is another cytoskeletal protein. The secretory vesicles do not migrate to the basolateral membrane if MEC is polarized. The apical membrane of MEC and the membrane of the secretory vesicle fuse, resulting in an opening through which the vesicle contents are discharged into the lumen of the alveoli.

### Synthesis of lactose

Glucose enters the MEC through its basolateral membrane via specific transporters (predominantly GLUT1) [63]. Some glucose is converted to galactose. Both glucose and galactose enter the Golgi to form lactose via a series of enzyme-catalyzed reactions. The formation of lactose in the Golgi results in the movement of water from the extracellular space (and thus the blood) into the cytoplasm and then into the Golgi where lactose ultimately becomes a component of milk. The Golgi apparatus is involved in processing of milk proteins, synthesis of lactose, and osmotic gradients for transport of water and, therefore, is very important in the synthesis of milk components. Lactose and much of the associated water in milk are released from secretory vesicles along with milk proteins into the lumen of the alveoli [76].

### Synthesis of fat

Precursors for synthesis of milk fats, including acetate, ß-hydroxybutyrate, acetoacetate, preformed fatty acids, glycerol, and monoacylglycerides, are taken up by the mammary gland [76]. The ketone bodies are important precursors of the synthesis of fatty acids in the milk of ruminants, rodents and humans, but not in pigs. This is because ketogenesis is limited in swine (including lactating sows) due to a deficiency of 3-hydroxy-3-methyl-glutaryl-CoA synthase in the liver. All of those components enter the pathway for synthesis of triglycerides in the smooth endoplasmic reticulum, leading to the formation of small lipid droplets. Numerous small lipid droplets fuse to yield larger droplets, which move toward the apical membrane. At the apical membrane, the large lipid droplet is exported out of the MEC through its apical membrane into the lumen of alveoli and the duct system of the mammary gland [63].

## Transport of milk components not synthesized in MEC

A number of components in maternal plasma are transported across MEC into the duct system essentially unchanged. These substances include immunoglobulins which bind to specific receptors on the basolateral surface of the MEC, enter the cell and are then exported into the lumen of the alveolus through the apical side of the cell via endocytic vesicles (or transport vesicles). In this process, the membrane of the transport vesicles fuses with the inner surface of the apical membrane of MEC and releases immunoglobulins into the lumen of the alveolus. As the transport vesicles traverse the cell, they do not seem to interact with the Golgi, secretory vesicles, or lipid droplets. Serum albumin may also be transported across MEC via this mechanism. Because there is no serum albumin receptor, serum albumin molecules are probably internalized into MEC along with immunoglobulins in the transport vesicles [48, 63].

### Paracellular pathway for nutrient transport

The tight junctions between MEC do not allow paracellular passage of macromolecules in the mammary epithelium, with the possible exception of water and ions. When the mammary gland is inflamed, such as during mastitis or involution, or when oxytocin is causing milk ejection, the tight junctions open to allow lactose and potassium to move from the lumen of the alveolus into the extracellular space and for sodium and chlorine to move into the lumen of the alveolus from the extracellular space. This results in a change in electrical conductivity by MEC, as well as increases in concentrations of lactose and other milk-specific components in blood. For instance, lactose can be measured in the urine of cows during the periparturient period. Milk proteins can be detected in the cow’s blood during lactation and early involution [63].

Other components of the blood that can enter the lumen of alveoli of the mammary gland via the paracellular pathway include leukocytes, which comprise the vast majority of somatic cells in milk [64]. Sodium, potassium and chloride are major monovalent ions in milk which are transported actively via Na^+^-K^+^ pump located on the basolateral membranes, but not the apical membranes, of mammary secretory cells. Ca^2+^, iron, zinc, copper, selenium, iodine, fluoride, and manganese are actively transported by MEC but the mechanisms remain poorly understood [110].

## Major milk proteins

All of the major milk proteins (except serum albumin and immunoglobulins) are synthesized from AA and secreted by MEC. The AA required for protein synthesis are either taken up from the blood or synthesized from numerous precursors in MEC [67]. Milk proteins consist of caseins (α-S1, α-S2, β, and κ) and whey proteins (α-lactalbumin, β-lactoglobulin, lactoferrin, γ-glubulin, and serum albumin) (Table 3). AA compositions of porcine proteins [111] are summarized in Table 4.

**Table 3:** Composition of proteins in mature milk of mammals

Protein	Cow	Pig	Sheep	Goat	Horse	Human
				g/L milk		
Caseins	28	28	46	29	13.6	4.4
α-S1	10.6	20	16.6	4.3	2.4	0.52
α-S2	3.4	2.4	6.4	5.8	0.2	0.0
β	10.1	2.3	18.3	11.2	10.7	2.8
κ	3.9	3.3	4.5	7.7	0.24	1.0
Whey proteins	6.0	20	10	5.0	8.3	6.6
α-Lactalbumin	1.2	3.0	2.4	0.85	2.4	2.8
β-Lactoglobulin	3.2	9.5	6.4	2.3	2.6	0.0
Lactoferrin	0.02–0.2	0.1–0.25	0.1	0.02–0.2	0.58	3–4
Transferiin	0.02–0.2	0.02–0.2	0.1	0.02–0.2	0.1	0.02–0.03
Immunoglobulins	1.1	6.6	0.4	0.4	1.6	1.2
Serum albumin	0.4	0.5	0.5	0.6	0.4	0.5

Adapted from Park and Haenlein [48] and Gallagher et al. [111]

**Table 4:** Composition of amino acids (residues per protein molecule) in caseins and whey proteins of porcine and bovine milk

AA	Porcine mlik						Bovine milk					
	Casein α-S1	Casein α-S2	Casein β	Casein κ	α-Lactalbumin	β-Lactoglobulin	Casein α-S1	Casein α-S2	Casein β	Casein κ	α-Lactalbumin	β-Lactoglobulin
Ala	7	8	10	15	3	13	9	8	5	15	5	19
Arg	10	3	6	9	2	5	6	6	4	5	1	3
Asp	6	5	5	2	15	14	7	4	4	3	13	10
Asn	8	12	5	10	6	4	8	14	5	8	8	5
Cys	0	2	0	1	8	4	0	2	0	2	8	7
Glu	21	28	18	12	5	14	26	24	19	13	7	16
Gln	22	18	23	10	6	8	14	16	20	14	7	10
Gly	10	2	5	2	8	3	9	2	5	2	7	5
His	11	6	5	3	3	3	5	3	5	3	4	2
Ile	6	16	9	12	9	6	11	11	10	13	9	10
Leu	16	8	26	4	11	24	17	13	22	8	17	27
Lys	11	25	13	8	11	11	14	24	11	9	12	16
Met	3	5	5	2	2	4	5	4	6	2	3	5
Phe	8	7	8	7	4	3	8	6	9	4	6	4
Pro	17	11	37	23	7	8	17	10	35	20	2	8
Ser	11	23	13	10	11	10	16	17	16	13	9	7
Thr	6	14	8	12	7	10	5	15	9	14	8	9
Trp	2	3	1	1	3	1	2	2	1	1	4	2
Tyr	10	12	5	9	4	2	10	12	4	9	4	4
Val	6	13	18	16	2	13	11	14	19	11	8	9
Total	191	220	218	168	127	160	200	207	209	169	142	178
P	8	17	6	1	x	x	8–9	12	5	1	x	x

Adapted from Gallagher et al. [111]

*P* = number of phosphate groups

### Caseins

Caseins are present in milk in the form of a micelle and can be phosphorylated by protein kinases in MEC. The phosphate groups covalently bound to the casein molecules bind calcium via ionic bonds. This initiates polymerization of the micelle particles and is essential for micelle formation. The casein micelle (140 nanometers in diameter) is a source of nutrients for neonates, supplying AA, calcium and phosphate. There are alpha, beta, gamma, and kappa caseins in milk [48]. Alpha-caseins are in multi-phosphorylated forms (s2, s3, s4, s5, and s6). Beta-casein is the major casein in the sow’s milk and is synthesized by MEC in response to prolactin [112]. Kappa-casein (a glycoprotein) is distributed throughout the casein micelle and stabilizes the micelle. Gamma-caseins are C-terminal fragments of ß-casein which are released after proteolytic degradation by plasmin when milk is within the alveoli of the mammary gland. Destabilization of the casein micelle by proteases is part of the mechanism involved in digestion of milk proteins in the stomach and intestine of neonates [113]. In lactating sows, the proportion of caseins in total protein of colostrum is ~ 9–32 % immediately after parturition and increases to ~ 30–45 % by 24 h postpartum, whereas the proportion of caseins in total protein of mature milk is ~ 50–60 % between d 7 and 28 of lactation [114].

### Major whey proteins

#### β-Lactoglobulin

This protein comprises of approximately 50 % of the total whey proteins in milk and it is the major whey protein in milk from ruminants and pigs [48]. β-Lactoglobulin is not found in the milk of many other species. The function of this protein is largely unknown, but it may be a fatty acid or lipid binding protein. β-Lactoglobulin has AA sequence similar to that for retinol-binding proteins [111]. Generally, β-lactoglobulin is found in the milk of mammals which transport high levels of immunoglobulins during colostrum formation; however, the specific relationship between the presence of β-lactoglobulin and immunoglobulin transport remains unclear. In lactating sows, concentrations of β-lactoglobulin in milk are relatively constant during the first 7 d of lactation (10–15 g/L) and decline thereafter to < 10 g/L on d 28 of lactation [114].

#### α-Lactalbumin

This protein accounts for about 25 % of the total whey proteins in milk and contains a high content of tryptophan. It is the regulatory subunit of the lactose synthase enzyme complex. Therefore, α-lactalbumin plays an important role in lactose production and secretion. α-Lactalbumin may have other nonspecific roles (e.g., binding Ca^++^ and Zn^+^) to affect the integrity of milk fat membranes [48]. Synthesis of this protein by MEC is induced by prolactin [112]. In lactating sows, concentrations of α-lactoglobulin are relatively low in colostrum (1.8 to 2 g/L), gradually increase to 3.3 g/L on d 7 of lactation, and decline moderately thereafter to ~ 3 g/L in mature milk [114].

### Minor whey proteins

#### Serum albumin

Serum albumin in milk is derived from maternal serum and is not synthesized in the mammary gland [48]. There are suggestions that this protein enters the milk via the paracellular pathway, or is taken up by MEC along with other components in serum such as immunoglobulins. There does not seem to be a specific mechanism for transport of serum albumin by MEC. Increases in the concentration of serum albumin in milk occur especially during mastitis and during involution of the mammary gland. The function of serum albumin in milk is unknown, but this protein can bind to long-chain fatty acids and some small molecules [48].

#### Immunoglobulins (Ig)

These proteins include IgG1, IgG2, IgA, and IgM. Immunoglobulins are present at very high concentrations in colostrum, but at much lower concentrations in mature milk [48]. For example, concentrations of IgG, IgA and IgM in porcine colostrum are 45–60, 3–5 and 3–5 times those in porcine mature milk, respectively (Table 5) [111]. Immunoglobulins provide passive immunity to offspring by being transported from the mother’s colostrum to the general circulation of the neonate. Immunoglobulins also serve as part of the immune system of the mammary gland. Secretory component (SC) is the part of the IgA receptor which is hydrolyzed by a protease, and remains attached to IgA during the process of secretion from the cell [48].

**Table 5:** Composition of immunoglobulins (Ig) in milk

Species	Type of milk	Ig A	Ig G	Ig M
			g/L	
Cow	Colostrum	3.9	50.5	4.2
	Mature milk	0.20	0.80	0.05
Goat	Colostrum	0.9–2.4	50–60	1.6–5.2
	Mature milk	0.03–0.08	0.1–0.4	0.01–0.04
Sheep	Colostrum	2.0	61	4.1
	Mature milk	0.06	0.3	0.03
Sow	Colostrum	10–26	62–94	3–10
	Mature milk	3.4–5.6	1.0–1.9	1.2–1.4
Human	Colostrum	17.4	0.43	1.6
	Mature milk	1.0	0.04	0.1

Adapted from Park and Haenlein [48] and Gallagher et al. [111]

### Other milk proteins

Lactoferrin (LF) is an iron-binding protein with antibacterial properties. LF is present at relatively low concentrations in milk from cows during lactation, but at relatively high concentrations when cows experience mastitis or involution of the mammary gland. Lactoferrin may also be an immunomodulatory protein, as it is the major nonspecific disease-resistance factor found in the mammary gland [63]. In lactating sows, concentrations of lactoferrin are ~ 1 g/L in colostrum, gradually increase to 0.3 g/L on d 7 of lactation, and decline further to 0.1–0.25 g/L in milk on d 28 of lactation [114].

Lactoperoxidase is a member of the heme peroxidase family of enzymes that break down hydrogen peroxide into water [12]. This protein also has antibacterial and anti-oxidant properties [63].

Lysozyme is an enzyme that cleaves the carbohydrate polymers of the bacterial cell wall to act as a bactericidal protein. This enzyme has a high activity in human milk and possibly horse milk, but its activity is relatively low in cow’s milk. Other enzymes in milk include proteases, protease activators, nucleases, glycosidases, AA oxidases, and D-amino acid oxidases [48]. These enzymes can degrade proteins, AA, D-amino acids, carbohydrates, and nucleotides in milk.

Beta 2-microglobulin was initially discovered as a crystalline precipitate in resuspended casein and was first called lactolin. It is part of the major histocompatability complex II (MHC II). While the function of beta 2-microglobulin in milk is not known, this protein is present in the epithelial cell in association with a protein that binds immunoglobulin G. Beta 2-microglobulin may be involved in function of the IgG receptor or in transport of IgG during colostrum formation [48].

## Milk protein synthesis and the role of mTOR

**Fig. 5:**
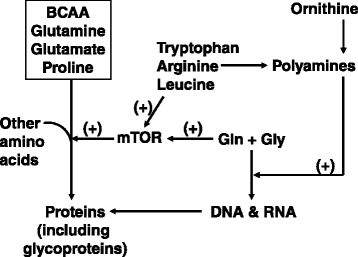
Roles of amino acids in the synthesis of milk protein by mammary epithelial cells. Amino acids are precursors of nucleic acids and building blocks of protein in mammary epithelial cells. In addition, certain amino acids can potentially activate mTOR signaling (the master regulator of mRNA translation) and are substrates for the synthesis of polyamines (substances that are essential for DNA and protein syntheses)

The major role of mTOR, particularly mTORC1 [mechanistic (mammalian) target of rapamycin complex 1], in the regulation of protein synthesis in animal cells has been well defined [115]. Protein synthesis is inhibited by the association of the nonphosphorylated eukaryotic translation initiation factor 4E binding protein 1 (4EBP1) with the eukaryotic translation initiation factor 4E (eIF4E), preventing the formation of the translation initiation complex. Activation of mTORC1 via protein phosphorylation phosphorylates 4EBP1 at multiple sites and subsequently causes the release of eIF4E from the 4EBP1-eIF4E complex [70]. Once released from this complex, eIF4E forms the active translation initiation complex with other translation initiation factors. The complete translation initiation complex then binds the 40S ribosomal subunit, generating the 43S pre-initiation complex which initiates translation of mRNA into protein. As noted previously, the mTORC1 is activated by certain AA, particularly leucine, arginine, glycine glutamine, and tryptophan [116–125] (Fig. 5). Additionally, leucine enhances overall protein synthesis by serving as a precursor for the synthesis of glutamate, glutamine, aspartate, asparagine, and protein [67–69]. At present, little is known about the regulation of mTOR and protein turnover in MEC.

During lactation, constitutive proteins are actively synthesized by cells of the mammary gland that may account for 40 to 70 % of total mammary protein synthesis [126]. In lactating sows, the synthetic rate for mammary constitutive proteins increases to a peak level on d 14 of lactation (7.8 g/kg wet tissue/d), and then decreases to 2.7 g/kg wet tissue/d on d 21 and to 0.5 g/kg wet tissue/d on d 28 of lactation [126]. There is evidence that dietary supplementation with arginine, which activates mTOR [70], enhances milk production by sows [127]. Similar results have been reported for dietary supplementation with leucine or a mixture of BCAA [128–132] (Table 6). As with other AA, antagonism among the three BCAA in diets should be avoided to achieve their desired effects [133]. Consistent with the findings from swine, duodenal infusion of leucine to lactating cows increased concentrations of casein, whey proteins, and total proteins in milk [134]. These results from animal studies have important implications for enhancing milk production in women who have impaired lactogenesis under various stressful conditions (e.g., premature birth, maternal complications, and high or low ambient temperatures) and in response to low intake of dietary protein [135].

**Table 6:** Effects of dietary BCAA supplementation to lactating sows on growth of suckling piglets

BCAA, Lys and CP content in basal diet (%)					BCAA supplementation (%)			Total BCAA in supplemental diet (%)			Milk DM yield	Litter weigh gain of piglets	Ref.
Leu	Ile	Val	Lys	CP	Leu	Ile	Val	Leu	Ile	Val			
1.36	0.5	0.72	0.9	14.5	0	0.35–0.7	0	1.36	0.85–1.2	0.72	↑	↑	[126]
1.36	0.5	0.72	0.9	14.5	0	0	0.35–0.7	1.36	0.5	1.07–1.42	↑	↑	[126]
0.95	0.58	0.61	0.8	14.2	0	0	0.26–0.37	0.95	0.58	0.77–0.98	x	NC^a^	[127]
0.95	0.58	0.61	0.8	14.2	0	0	0.26–0.37	0.95	0.58	1.15	x	NC^b^	[127]
0.95	0.58	0.61	0.8	14.2	0	0	0.26–0.37	0.95	0.58	1.15	x	↑^c^	[127]
0.95	0.58	0.94	1.2	20.5	0	0	0.23–43	0.95	0.58	1.17–1.37	x	NC^d^	[127]
0.95	0.58	0.94	1.2	20.5	0	0	0.23–43	0.95	0.58	1.17–1.37	x	NC^e^	[127]
0.95	0.58	0.94	1.2	20.5	0	0	0.23–43	0.95	0.58	1.17–1.37	x	↑^f^	[127]
1.31	0.64	0.75	0.90	14.3	0	0	0.1–0.4	1.31	0.64	0.85–1.15	x	↑	[128]
1.57	0.68	0.8	0.9	15.5	0.4	0	0	1.97	0.68	0.8	NC	NC	[129]
1.57	0.68	0.8	0.9	15.5	0	0.4	0	1.97	1.08	0.8	NC	NC	[129]
1.57	0.68	0.8	0.9	15.5	0	0	0.4	1.97	0.68	1.2	NC	↑	[129]
1.18	0.65	0.45	1.01	15.5	0	0	0.1–1.0	1.18	0.65	0.55–1.45	↑	↑	[130]

^a^All sows with the average total number of pigs weaned/litter being 9.8 to 10.0

^b^Sows with the average total number of pigs weaned/litter being 8.61 to 8.91

^c^Sows with the average total number of pigs weaned/litter being 10.4 to 10.6

^d^All sows with the average total number of pigs weaned/litter being 9.95 to 10.0

^e^All sows with the average total number of pigs weaned/litter being 8.7–8.91

^f^All sows with the average total number of pigs weaned/litter being 10.5

*NC* no effect; ↑: Increase; The sign “x” denotes the lack of data

## Conclusion and perspectives

Milk is of supreme importance to survival, proper development, and dynamic growth of the neonate. Furthermore, lactation is an integral component of the highly successful reproductive strategy of mammalian species. In fact, development of the mammary gland, which is affected by the provision of AA, is closely linked to the reproductive cycle of all mammalian species. The synthetic capacity of the mammary gland depends largely on the number and efficiency of functional MEC and directly correlates with subsequent growth of suckling neonates. In the lactating mammary gland, BCAA are extensively transaminated with α-ketoglutarate to produce glutamate and BCKA, with glutamate being an essential substrate for synthesis of several AA (glutamine, alanine, aspartate and asparagine). Moreover, some of the BCAA carbons are either oxidized to provide ATP in mitochondria or utilized for lipid synthesis in the cytosol. Leucine, glutamine and arginine stimulate the mTOR cell signaling pathway to enhance protein synthesis and milk production. At present, there is a paucity of information about the metabolism of AA other than BCAA and arginine in the lactating mammary tissue or MEC. For example, it is unknown whether a severe deficiency of glycine in the milk of species (e.g., pigs, cows, and humans) [120] results from inadequate uptake of this AA by MEC or its extensive catabolism in the cell. Because milk production by farm mammals (e.g., sows [66, 79] and cows [63, 136]) is affected by both nutritional and hormonal factors, research on AA metabolism in mammary tissue will not only advance our knowledge of lactation biology, but will also have practical implications for improving the efficiency of livestock production to sustain animal agriculture worldwide [137–141]. Results from animal studies will also have important implications for optimizing milk synthesis by lactating women as well as infant growth and development in both developed and developing countries.
